# Deep-learning-based instrument detection for intra-operative robotic assistance

**DOI:** 10.1007/s11548-022-02715-y

**Published:** 2022-07-28

**Authors:** Jorge Badilla-Solórzano, Svenja Spindeldreier, Sontje Ihler, Nils-Claudius Gellrich, Simon Spalthoff

**Affiliations:** 1 Institute of Mechatronic Systems, Leibniz University Hannover, Hannover, Germany; 2 Department of Cranio-Maxillofacial Surgery, Hannover Medical School, Hannover, Germany

**Keywords:** Robotic scrub nurse, Dataset, Data augmentation, Robot-assisted surgery, Mask R-CNN, Mask-based object insertion

## Abstract

**Purpose:**

Robotic scrub nurses have the potential to become an attractive solution for the operating room. Surgical instrument detection is a fundamental task for these systems, which is the focus of this work. We address the detection of the complete surgery set for wisdom teeth extraction, and propose a data augmentation technique tailored for this task.

**Methods:**

Using a robotic scrub nurse system, we create a dataset of 369 unique multi-instrument images with manual annotations. We then propose the Mask-Based Object Insertion method, capable of automatically generating a large amount of synthetic images. By using both real and artificial data, different Mask R-CNN models are trained and evaluated.

**Results:**

Our experiments reveal that models trained on the synthetic data created with our method achieve comparable performance to that of models trained on real images. Moreover, we demonstrate that the combination of real and our artificial data can lead to a superior level of generalization.

**Conclusion:**

The proposed data augmentation technique is capable of dramatically reducing the labelling work required for training a deep-learning-based detection algorithm. A dataset for the complete instrument set for wisdom teeth extraction is made available for the scientific community, as well as the raw information required for the generation of the synthetic data (https://github.com/Jorebs/Deep-learning-based-instrument-detection-for-intra operative-robotic-assistance).

## Introduction

**Fig. 1:**
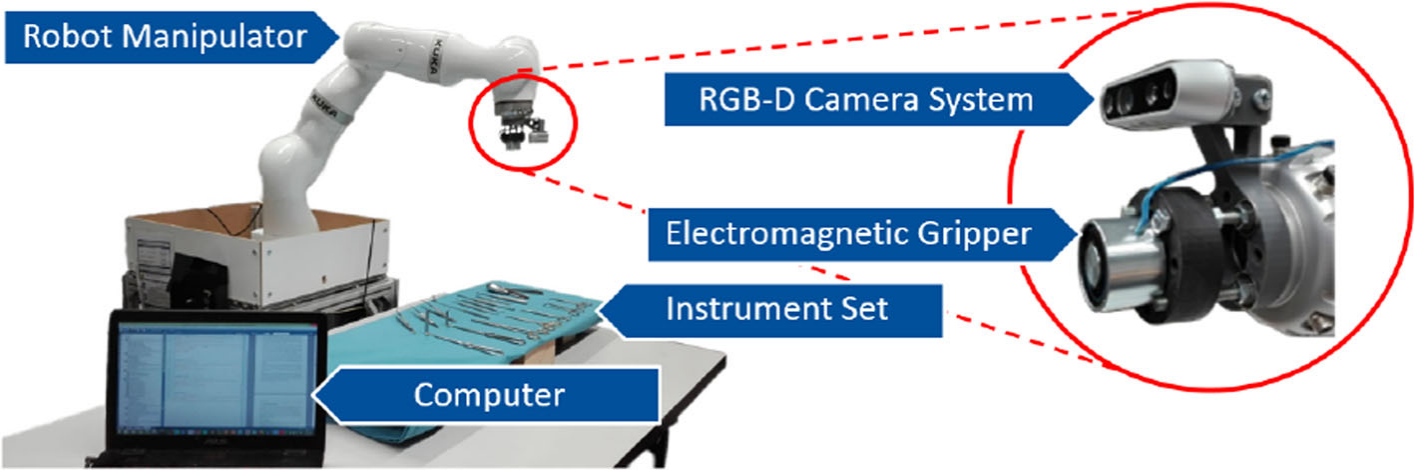
Complete robotic scrub nurse system used during our experiments. The system relies on a robot manipulator, an RGB-D camera system, a computer, and a gripper to interact with surgical instruments located on a medical table

Robot-assisted surgery has gained significant relevance in the last few years. Improvements in safety and efficiency during surgical procedures and the reduction of the probability of infection, make robot-assisted surgery an attractive solution for medical centers [22]. Robots can not only be involved directly in surgical procedures but also play a meaningful supporting role in the operating room. A clear example of this are robotic scrub nurses (RSNs), systems responsible for handing surgical instruments to the operating physician and retrieving them after their use. An RSN has the potential of making great contributions to the medical field since it could support surgeons during staff shortages and become an economically attractive system. An example of an RSN system is presented in Fig. 1.

A fundamental task of an RSN is to detect and identify surgical instruments within a set. Thus, there is a need for accurate surgical tool detection techniques that allow the RSN to reliably retrieve a requested instrument upon command. This can be observed in the numerous related studies that focus on instrument detection [1, 15, 16].

The most popular solution for the detection task is to utilize a camera. Among the camera-based approaches found in the literature is the work presented in [21], which relies on the use of markers attached to the instruments, and also the works of Zhou et al. [23, 24] which identify instruments one at a time, employing an identification tray. Similarly, in [16], an RGB-D camera and 3D-printed models of surgical instruments are employed during the experiments.

Finding surgical instruments in images constitutes an application of object detection/segmentation. State-of-the-art supervised deep-learning algorithms are hence a potential solution. These techniques require annotated datasets that would ideally include the corresponding segmentation masks since they not only provide information regarding the instruments’ location but also their geometry, which can be relevant for a gripping task. To the best of our knowledge, there is no publicly available image dataset for a complete surgical instrument set that includes the ground-truth segmentation masks.

In general, bigger datasets achieve higher accuracy and better generalization properties of a trained deep-learning model. Hence, a large amount of annotated data is desirable. Nevertheless, the labelling process is time-consuming and expensive. For this reason, data augmentation (DA) techniques are popular since they can produce synthetic automatically annotated data. Advanced augmentation methods are based on the segmentation of objects of interest as foreground and the replacement of their background with specific image content. Examples of this are presented in [9, 19]. In [5], the authors apply a cut-and-paste technique and demonstrate how the insertion of objects of interest onto different background images constitute a useful way of generating reliable synthetic data.

In this work, our main goal is to successfully detect the instruments of a complete surgical set from camera images without relying on external means (e.g., barcodes, stickers, predefined positions) and by considering the presence of multiple instruments in the scene. For this matter, the complete instrument set for wisdom teeth extraction surgery (18 instruments) is chosen and the deep-learning algorithm Mask R-CNN [6] is applied. To reduce the amount of required manually-labelled data, we introduce a DA approach, called Mask-Based Object Insertion (MBOI). This technique takes inspiration from the method presented in [5] and exploits the way the instruments are set up on the instrument tray (on a flat surface, with a similar looking background and arranged in an orderly way so that occlusions are avoided). The MBOI method utilizes a reduced amount of annotated single-instrument images and the depth information of the scene to generate a large amount of automatically annotated multi-instrument images, in which the relative size of the instruments is preserved. Our approach is therefore introduced to address the need for reliable surgical tool detection and promote further research in the field of RSNs.

The contributions of our work are: 1) the creation and publication of a labelled image dataset for the complete instrument set for wisdom teeth extraction surgery with multi-instrument images and including the corresponding segmentation masks, 2) the introduction of a data augmentation method based on the cut-and-paste technique for object detection/instance segmentation, especially useful when both the RGB images and depth maps of the scene are available, 3) the additional creation and publication of a collection of single-instrument images of the instrument set with corresponding segmentation masks and depth maps, 4) deep-learning-based multi-instrument detection of a complete surgical instrument set under quasi-real conditions, similar to those found in the operating room.

## Related work

Several research groups have studied the performance of an RSN. However, in these studies, important simplifications are considered that deviate from the actual surgical scene. An example of this is presented in [14], where the authors recognize the challenges associated with the use of multiple instruments and hence, limit their prototype to exclusively interact with an endoscope. In [7, 8], one of the most well-known works in the field of RSNs is presented, namely the *Gestonurse*. The authors develop a multi-modal operation, allowing the system to retrieve five different instruments by interpreting both gestural and oral commands. The instruments’ positions are predefined, and no object detection is applied.

Other RSN-related works have greater focus on the identification of surgical instruments. In [2], a robotic system capable of interpreting voice commands, identifying different surgical instruments, and grasping them using an electromagnetic gripper is proposed. However, a limited set of only seven instruments is employed. Additionally, the authors rely on the Matrox Imaging Library [13] for the instrument identification, which is a non-open-source image processing software. Another interesting approach is provided in [23, 24], where the identification of five different instruments is addressed by grasping an unidentified instrument, putting it into an identification tray and using machine learning to determine its class. This is a slow and unreliable procedure for an intra-operative application of an RSN.

The instrument identification based on external means is studied in [21], where the authors rely on small barcodes attached to the instruments and a scanner to determine each instrument’s class. This requires high resolution images and high proximity, as well as previously prepared instruments and visible barcodes during the identification process, which can increase the cost of the system, the identification time, and be susceptible to errors produced by overexposure.

## Dataset

A suitable dataset is fundamental for a deep-learning-based RSN. Therefore, the setup in which the dataset is created must resemble the actual conditions of an operating room and contain enough information so that a trained deep-learning algorithm is capable of achieving good generalization on unseen data.

### Robotic scrub nurse system

With the goal of creating an image dataset useful in a real RSN application, an actual RSN system (see Fig. 1) is employed for the image generation. The system was constructed in our research lab and it includes a 7-degree-of-freedom robotic arm (KUKA LBR iiwa 14 R820), an RGB-D camera system (Intel$^\circledR$ RealSense$^{\mathrm{TM}}$ D435) in a hand-in-eye configuration, a complete instrument set, an electromagnetic gripper, and a computer (GPU: Nvidia GeForce GTX 980Ti), where the programming was implemented using the Python API Keras based on Tensorflow 1.13.1.

### Instrument set

The selected instrument set corresponds to the surgical set for wisdom teeth extraction. This set has a total of 18 different instruments. Despite the relatively small number of instruments, the set includes both similar-looking instruments and others that differ significantly in both shape and size. The great variety and limited number of instruments make this set ideal for our application.

**Fig. 2:**
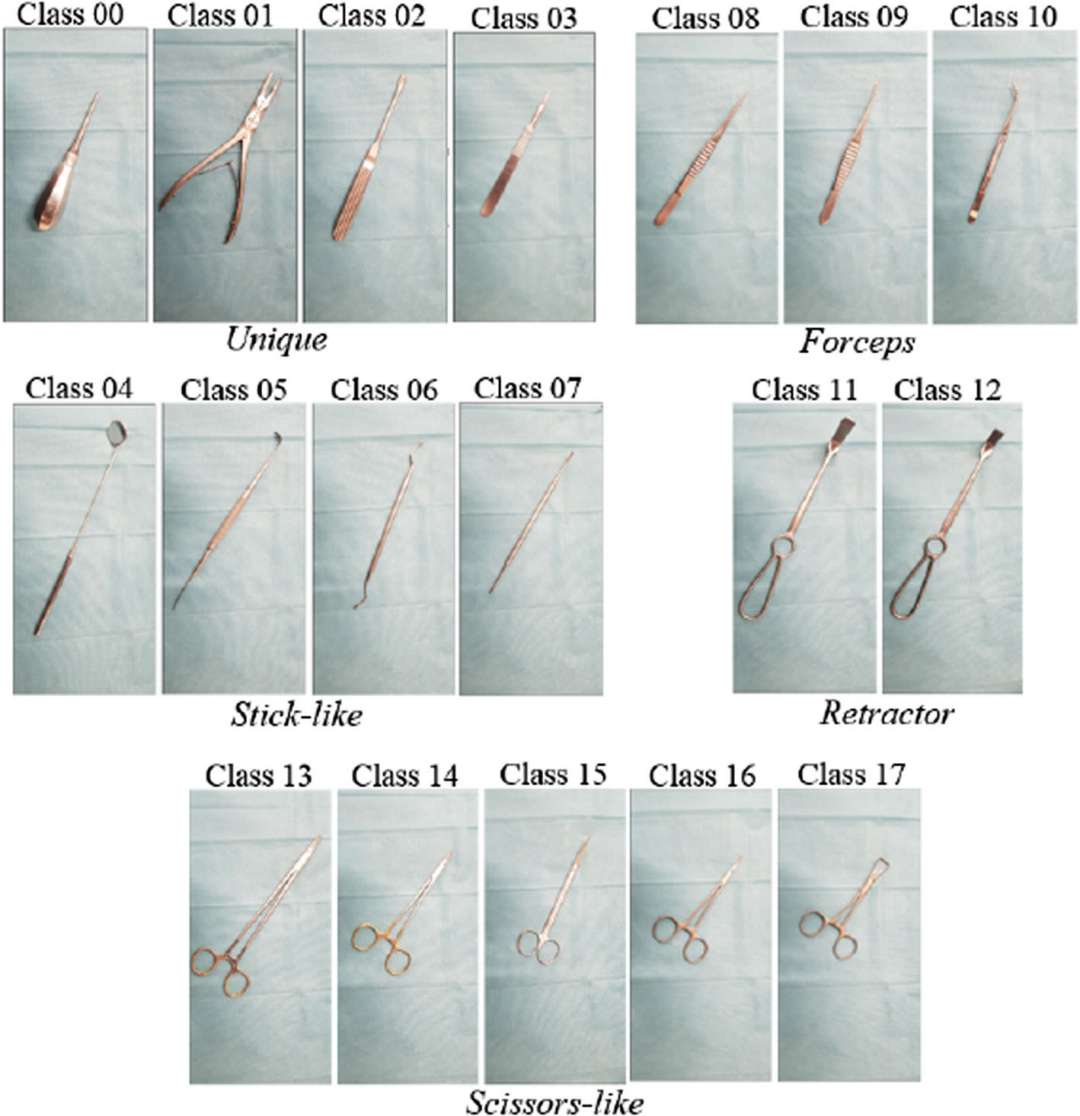
18 instruments of the surgical set for wisdom teeth extraction and their corresponding class labels. The instruments are classified based on their shape in the groups *Unique*, *Stick-like*, *Forceps*, *Retractor*, and *Scissors-like*

Similar-looking instruments are expected to lead to poor performance of the detection algorithm. In order to better study this potential challenge, the instruments are classified according to their shapes. The different shape types are: *Unique*, *Stick-like*, *Forceps*, *Retractor*, and *Scissors-like*. The names, shape type, and class designation for the detection algorithm are presented in Table 1. Additionally, Fig. 2 shows pictures of all the instruments in the surgical set.

**Table 1:** Instrument names, shape types, and classes of the instrument set for wisdom teeth extraction

Instrument name	Shape type	Class
Root elevator	Unique	Class 00
Dental pliers	Unique	Class 01
Raspatory	Unique	Class 02
Scalpel holder	Unique	Class 03
Dental mirror	Stick-like	Class 04
Freer raspatory	Stick-like	Class 05
Dental sharp spoon	Stick-like	Class 06
Luniatschek gauze packer	Stick-like	Class 07
Anatomical forceps	Forceps	Class 08
Surgical forceps	Forceps	Class 09
Dental forceps	Forceps	Class 10
Long retractor	Retractor	Class 11
Short retractor	Retractor	Class 12
Big needle holder	Scissors-like	Class 13
Small needle holder	Scissors-like	Class 14
Surgical Scissors	Scissors-like	Class 15
Surgical clamp	Scissors-like	Class 16
Backhaus towel clamp	Scissors-like	Class 17

### Images and annotations

During the generation of our dataset, the instruments are set up on a surgical cloth in such a way that inter-instrument occlusions are avoided. This is meant to depict the real-world scenario, in which the instruments are arranged on a tray in an orderly fashion. The RGB-D camera mounted on the robot’s end-effector is used to generate the images. Different end-effector poses are used to simulate the actual operation of the RSN, while the instruments are rearranged regularly to increase the spatial variability of the data. This means that the size of the instruments varies from one image to another. A total of 369 images, with a resolution of 640x480 pixels, are created. The generated images are manually annotated using the online tool available at [4], obtaining the segmentation masks for each image. The corresponding bounding boxes are determined on the basis of these masks.

This labelled dataset is split into disjoint training, validation, and test sets. The test and validation sets contain 72 images each. The remaining 225 images form the base of the training set. To increase the number of the training samples, the images are artificially augmented using horizontal, vertical, and diagonal flips creating a total of 900 annotated images in the training set.

## Mask-based object insertion

A trained deep-learning model relies on the quality of the dataset with which it is trained. The generalization capacity of a model increases with the size of its training set. A common approach to artificially increase the size of a training set is data augmentation (DA) [3, 10, 18].

Simple DA techniques rely on the application of geometric and color transformations to the manually annotated images to generate artificial data. Some more advanced augmentation techniques are based on the segmentation of objects of interest as foreground and the replacement of their background with specific image content. Examples of this are presented in [9, 11, 17, 19].

Inspired by this concept, we introduce a segmentation-based DA approach, called Mask-Based Object Insertion (MBOI). The MBOI method can automatically generate synthetic multi-instrument (MI) images from annotated single-instrument (SI) images, while creating their corresponding bounding boxes and segmentation masks. For this, the method employs a collection of annotated SI images per instrument class, their corresponding depth maps, and a collection of background images. In our case, background images simply correspond to pictures of the surgical cloth under different illumination conditions and camera angles. The background and SI images, as well as the corresponding depth maps are created using the robot-mounted camera presented in Fig. 1, using different end-effector poses. This implies that the size of the instruments is different in every image and in some cases a small part of the instrument is out of the scene.

### Synthetic multi-instrument images

**Fig. 3:**
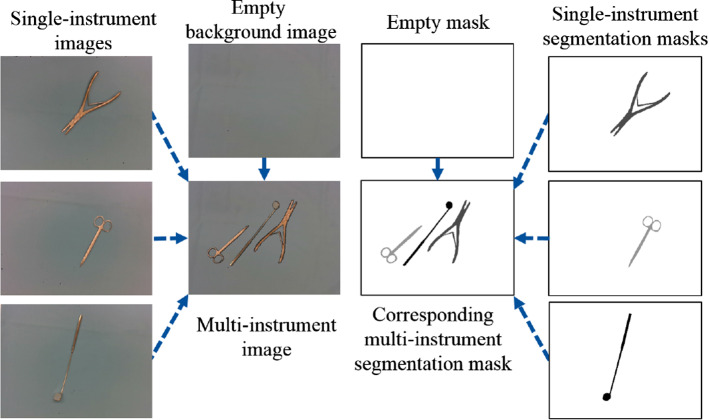
Diagram of the MBOI method for three different instruments. The background of the masks is set as white for a better visualization. The instruments and their corresponding segmentation masks are cut and pasted onto the background images one by one. The 3D information corresponding to the single-instrument images is used to preserve the relative size of the instruments in the synthetic multi-instrument images and the corresponding masks

For the creation of each synthetic image, a background image is randomly selected, as well as a subset of instrument classes. For each instrument class in this subset, an SI image is chosen from its corresponding collection. A randomized spatial transformation is then applied to each of the SI images and their corresponding masks, leading to a different transformation for each class. The instruments are then segmented as foreground using their segmentation masks and inserted one by one onto the previously selected background image, by substituting the corresponding pixel values, to create a synthetic MI image. Similarly, the transformed masks are inserted into a black single-channel image, creating the corresponding segmentation mask. A diagram of the method is presented in Fig. 3, where a background image, three SI images, and their segmentation masks are employed to generate an MI synthetic image and its corresponding mask. The spatial transformations applied on the SI images significantly increase the variability on the generated synthetic data, which is expected to lead to better performance of the trained model.

The MBOI method makes the following considerations: 1) The scale of the first inserted instrument is the same as that of SI image, while the scale of the others is calculated so that the relative size of the instruments is preserved. 2) The method keeps count of the amount of instances introduced in the generated data and guarantees that the classes in the synthetic MI images are balanced. 3) In typical instrument arrangements, the instruments are set up in a way such that overlapping is avoided. Hence, the pixel insertion is carried out in such a way that no overlapping takes place. 4) If after the insertion onto the background image a considerable part of the original instrument from the SI image is out of frame, the instrument is not annotated since it is considered that the synthetic MI image does not contain enough information for the determination of its class.

### Spatial transformation

The randomized spatial transformation is determined by the parameterized 2D homogeneous transformation matrix $\mathbf {T}$, given by:

1 $$\begin{aligned} \mathbf {T}= \begin{pmatrix} s \left( -1 \right) ^{n} \cdot \cos \theta &{} -\sin \theta &{} t_{x}\\ \sin \theta &{} s \left( -1 \right) ^{m}\cdot \cos \theta &{} t_{y}\\ 0 &{} 0 &{} 1\\ \end{pmatrix} \end{aligned}$$

with $n,m\sim \mathrm {Bernoulli}(0.5)$, $\theta \sim \mathrm {Uniform}(-\frac{\pi }{2},\frac{\pi }{2})$, $t_{x}\sim \mathrm {Uniform}(-\frac{w}{2},\frac{w}{2})$, $t_{y}\sim \mathrm {Uniform}(-\frac{h}{2},\frac{h}{2})$, where *w* and *h* correspond to the width and height of the image, in that order. In this transformation, *n* and *m* determine the flips of the image, while $\theta$ determines its rotation. $t_x$ and $t_y$ correspond to the horizontal and vertical displacements. Finally, the parameter *s* corresponds to the scale factor, which is especially interesting and is explained in the following subsection.

### Preservation of the relative size

Since the SI images are created with different distances between the camera and the instrument, the size of the instruments is different in each of them. If the MBOI method were to be applied without taking the size of the instruments into consideration, the MI images would include instruments whose relative size would not match the reality.

In order to preserve the relative size of the instruments in the synthetic MI images, the MBOI method relies on the concept of scale. In this work, the scale *s* of an instrument in an specific image is defined as the quotient of its image length $l_\mathrm {{[p]}}$ (maximum length of an instrument in an image, obtained from their segmentation mask), measured in pixel units, and its physical length $l_\mathrm {{[m]}}$ (determined by the 3D information from the corresponding depth map), measured in meters.

When creating an MI image, after applying the spatial transformation of the first instrument foreground to be inserted, the instrument’s scale is calculated and taken as reference ($s^0$) for the subsequent instrument foregrounds to be inserted. The insertion is then made in such a way, that the relative size of the instruments in the MI images is preserved. This is done by applying a scale $s^i$ given by:

2 $$\begin{aligned} s^i = \frac{l^i_\mathrm {[m]}}{l^i_\mathrm {[p]}} \cdot s^0 = \frac{l^i_\mathrm {[m]}}{l^i_\mathrm {[p]}} \cdot \frac{l^0_\mathrm {[p]}}{l^0_\mathrm {[m]}} \end{aligned}$$

The relative size of the instruments is considered a meaningful feature for their identification since our instrument set includes similar-looking instruments, whose main difference is their size. Clear examples of this can be seen in the scissors-like instrument group for the big and small needle holders (Class 13 and Class 14), as well as the surgical clamp (Class 16), as shown in Fig. 2).

### Occlusion avoidance

During the creation of the synthetic MI images the inserted instruments can potentially overlap each other. The MBOI approach detects such events and avoids them by re-sampling the parameters of the transformation matrix $\mathbf {T}$ and applying it to the SI images until no overlapping takes place. If certain number of iterations is reached (20, in our experiments) and the issues persist, the instrument in question is omitted from the final MI image.

### Instances partially out of frame

If a large portion of an instrument is missing from an image, the determination of the instrument’s class can become impossible, even for a human observant. The MBOI method takes this into account by not annotating instruments in cases where at least 50% of the instrument’s pixels are missing in the synthetic MI image after the insertion.

## Experiments and results

In order to apply the MBOI method and use the synthetic MI images in our experiments, 20 background images, 12 SI images per instrument class, as well as their corresponding depth maps, are generated using the RGB-D camera in the robot’s end-effector (see Fig. 1). The SI images are then annotated. All of these data are made publicly available, along with the 369 real images, to support further research regarding medical instrument detection: https://github.com/Jorebs/Deep-learning-based-instrument-detection-for-intra-operative-robotic-assistance.

Employing the aforementioned data, the MBOI technique, and the 900 annotated images mentioned in Sect.“Images and annotations” , four different Mask R-CNN [6] models are trained:

1. **Baseline-model**: Trained using the 900 images (225 manually annotated images plus their flips).
2. **Pseudo-MBOI-model (PMBOI-model)**: Trained on a fully-synthetic dataset created with the MBOI method without preserving the relative size of the instruments. This dataset includes a total of 2892 images and 1000 instances per instrument, with a minimum and maximum number of instruments per image of 3 and 12, respectively.
3. **MBOI-model**: Trained on a fully-synthetic dataset created with the MBOI method preserving the instruments’ relative size. This dataset includes a total of 2940 images and 1000 instances per instrument, with a minimum and maximum number of instruments per image of 3 and 12, respectively.
4. **MBOI+-model**: Trained on a dataset that combines the 2940 images of the MBOI-model as well as the 900 images of the baseline-model.

The training weights of the COCO dataset [12] are used as initialization weights for all training processes. Additionally, the resolution of the images is set to 512x512 pixels. The training is performed using batch size of 3 and a learning rate of 0.0001, using stochastic gradient descent with momentum as optimizer. Both the validation and test sets remain as defined in Sect. “Images and annotations” throughout these experiments. The models are trained until convergence. Finally, all instrument detections with class scores below 0.7 are discarded, and therefore not included in the results.

### General performance of the models

**Fig. 4:**
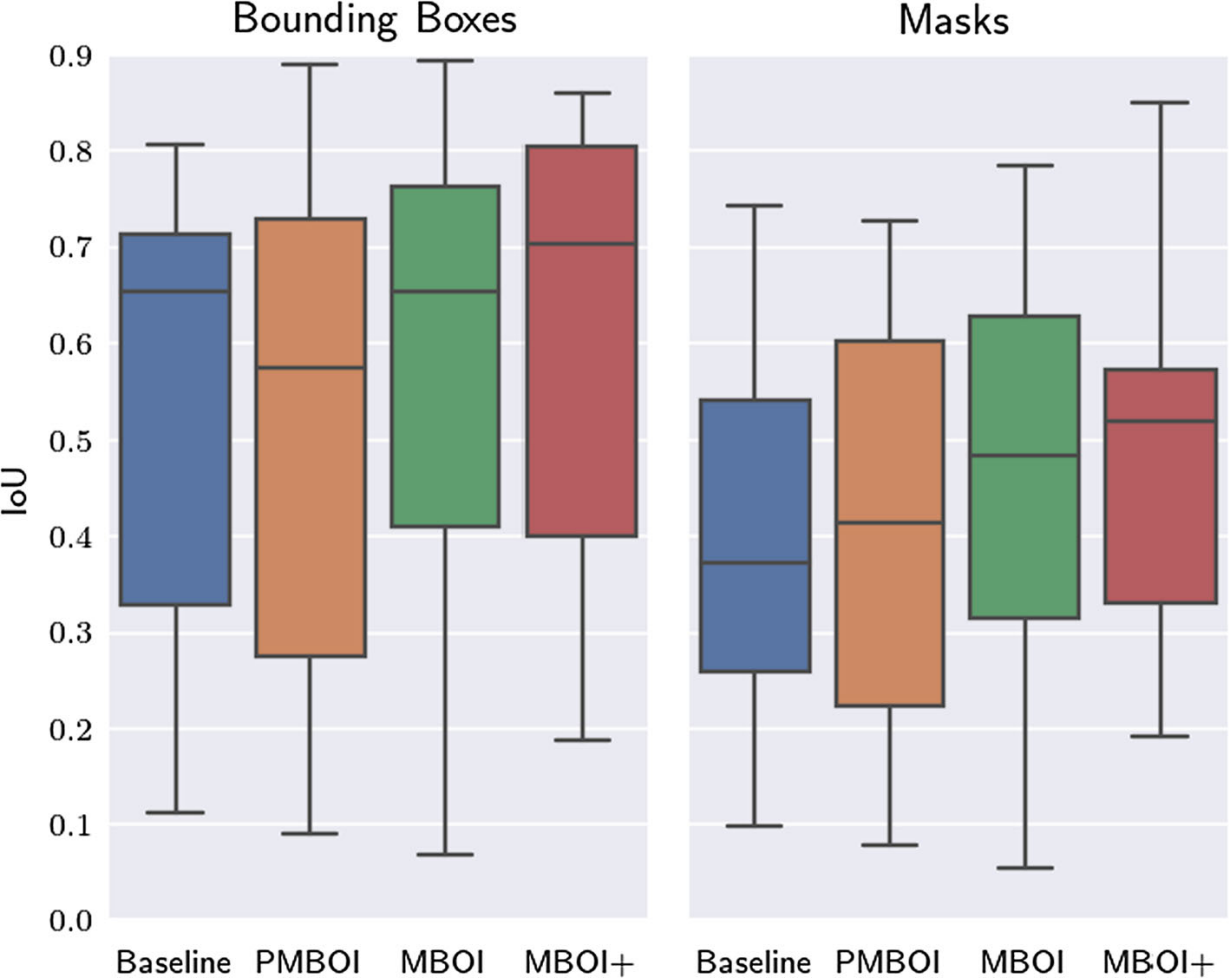
Boxplots of the IoU values for the baseline-model (blue), pseudo-MBOI-model (orange), MBOI-model (green), and MBOI+-model (red). All models are evaluated on the test set. Qualitatively, the results indicate a better performance for the MBOI-model in comparison to the pseudo-MBOI-model. The results also suggest that the MBOI+-model is superior to all the others

**Fig. 5:**
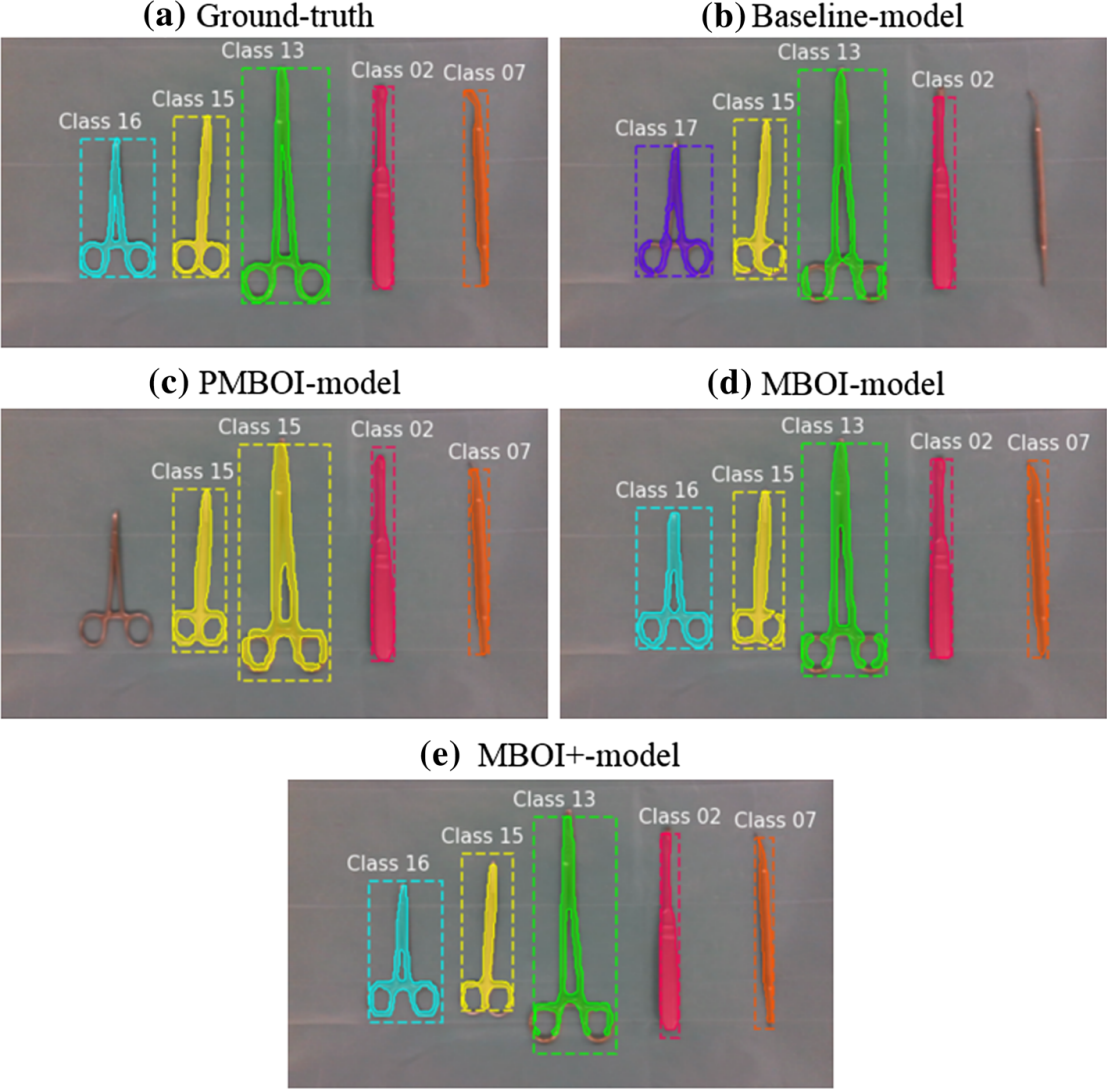
Qualitative examples of the performance of the trained Mask R-CNN models. The predictions include the instrument class, the bounding boxes, and segmentation masks. Each instrument class is assigned a fixed color for the corresponding bounding boxes and masks. The expected output is presented in the ground-truth (a). In this particular example, the baseline-model (b) mis-classifies Class 16 for Class 17 and Class 07 as part of the background, while the pseudo-MBOI-model (c) mis-classifies Class 13 for Class 15 and fails to detect Class 16. Very accurate results are obtained by the MBOI-model (d) and the MBOI+-model (e), being close to identical to the ground-truth. It is important to note, that this is just a qualitative example and does not necessarily reflect the average behavior of the trained models

To compare the general performance of all the models on the test set, the Intersection over Union (IoU) metric is employed. This metric is applied on both the bounding boxes and masks and the results are shown in Fig. 4. A qualitative example of the performance of each model is presented in Fig. 5

Figure 4 suggests some interesting conclusions. First, the MBOI+-model seems to outperform the others for both the bounding boxes and the segmentation masks. Additionally, the MBOI-model appears to lead to higher IoU values than those corresponding to the pseudo-MBOI-model, potentially indicating the relevance of the preservation of the relative instrument size during the application of the MBOI technique. Another potential conclusion is that the MBOI-model leads to comparable results to those of the baseline-model, despite being trained entirely on artificial data, created using only 12 manually annotated instances per instrument class (corresponding to the SI images).

**Table 2:** Mean values of the IoU (mIoU) metric for the trained Mask R-CNN models, evaluated on the test set

	Baseline	PMBOI	MBOI	MBOI+
**mIoU**$_{bbox}$	0.555	0.543	0.576	0.682
**mIoU**$_{masks}$	0.375	0.388	0.435	0.520

**Fig. 6:**
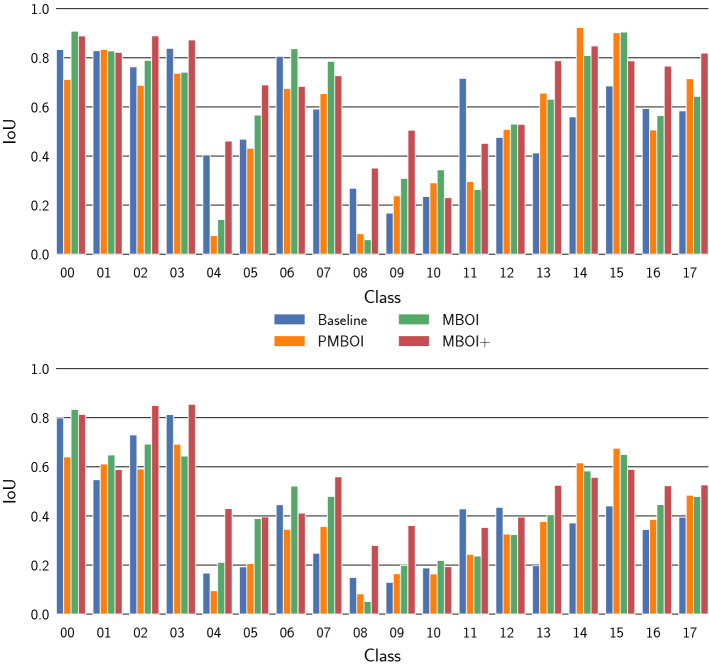
Average IoU values per instrument class for the baseline-model, the pseudo-MBOI-model, the MBOI-model, and the MBOI+-model. The presented results are obtained from the evaluation on the test set. Top: Values for the bounding boxes, Bottom: Values for the masks

Dependent Student’s t-tests for paired samples are then performed to verify the statistical significance of these potential conclusions. For this matter, intervals of $0.05 \le p < 0.10$, $0.01 \le p < 0.05$, and $0.00 \le p < 0.01$ are selected for weak, moderate, and strong significance, respectively.

With the p-values of $p_{\text {bbox}} = 0.077$ and $p_{\text {mask}} = 0.053$, for the case of the pseudo-MBOI-model and the MBOI-model, the t-test indicates a weak statistical significance for the performance difference, for both the bounding boxes and masks. When analysing the difference between the performances of the baseline-model and the MBOI+-model, the p-values correspond to $p_{\text {bbox}} = 0.091$ and $p_{\text {mask}} = 0.007$, which indicate a weak statistical significance for the case of the bounding boxes and a strong significance for the case of the segmentation masks. Finally, the application of the t-test for the performance difference for the baseline-model and the MBOI-model leads to p-values of $p_{\text {bbox}} = 0.690$ and $p_{\text {mask}} = 0.167$. Since both values are greater than 0.10, it can be concluded that there is no evidence of a statistically significant difference for both models’ performances. This is a relevant conclusion, since it indicates that training using synthetic data generated via the MBOI method can lead to similar performance to that of models trained on real data, with significantly less amount of annotating work (12 instances per instrument class).

To further analyse the performance of the models, the average values of the IoU metric for each of them are presented in Table 2. The values clearly suggest that the MBOI+-model is the most suitable for the detection task, followed by the MBOI-model. Despite the weak statistical significance observed when comparing the PMBOI-model and MBOI-model, the latter presents, in average, a higher performance, indicating that the preservation of the relative size in the synthetic images leads to better results. Additionally, the contribution of the data generated with the MBOI method is clear, when comparing the performance of the baseline and MBOI+-model.

### Average performance per instrument class

In order to understand the performance of the models on each instrument class, the average values of the IoU metric for both the bounding boxes and the masks are presented in Fig. 6.

This figure indicates that the IoU values for the dental mirror (Class 04), the forceps (Class 08, Class 09, and Class 10), and the retractors (Class 11, and Class 12) are considerably lower than rest in most if not all cases. This can be attributed to the fact that all of these instruments share their shape type with other instruments. This can not only cause mis-predictions between instruments of the same shape type, but also reduce the class score of a correct prediction, which can lead to the detection being discarded and being wrongly classified as background. To further analyze this, the confusion matrix for the detected instruments can be studied.

### Analysis of mis-classifications

**Fig. 7:**
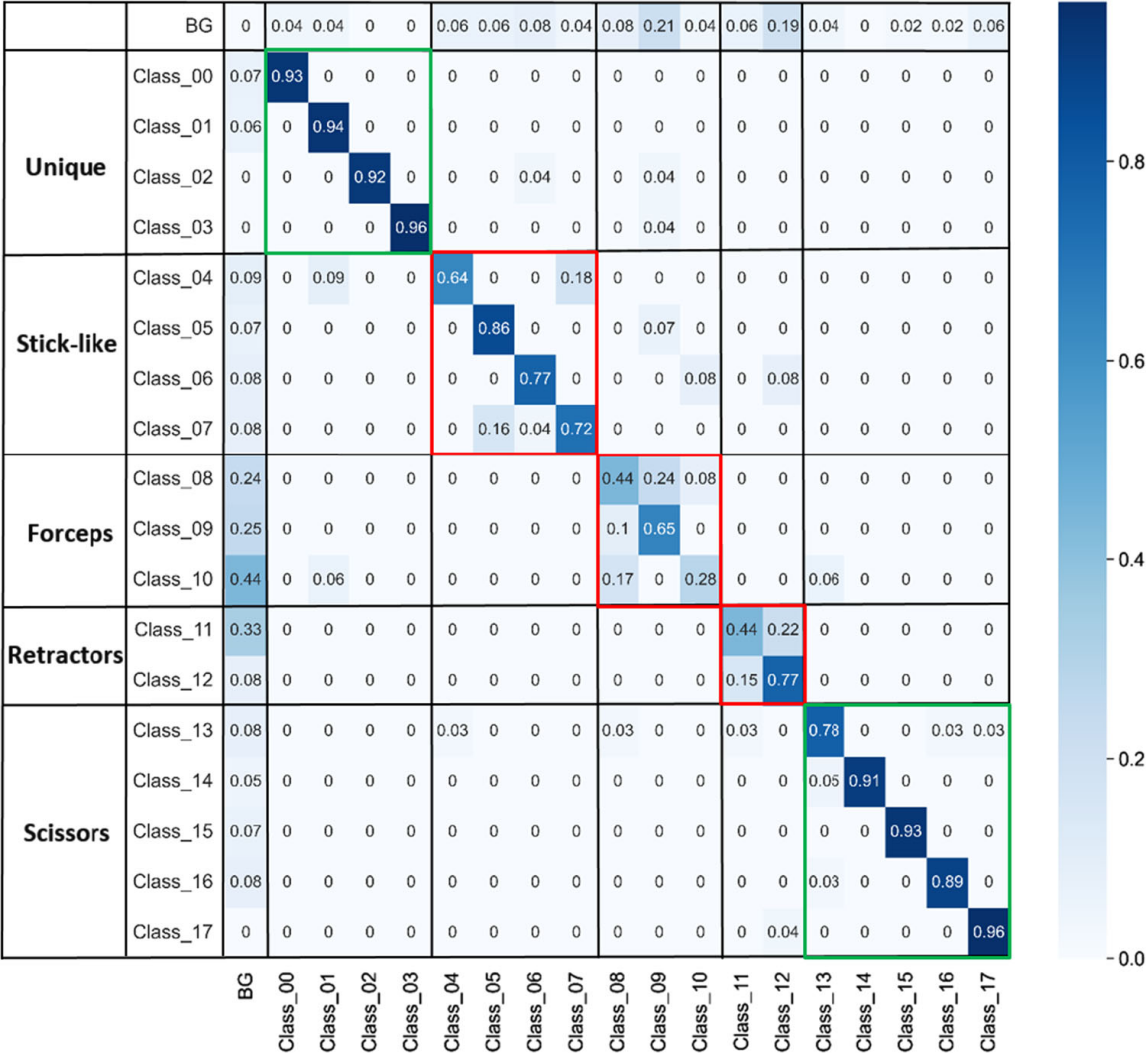
Normalized confusion matrix of the MBOI+-model evaluated on the test set. The areas of the confusion matrix corresponding to predictions of instruments belonging to the same shape type are highlighted. Areas of high performance are assigned the color green, while areas of poor performance are highlighted in red. Entries on the main diagonal of the confusion matrix higher or equal to 0.70 are considered indicators of high performance, while lower values are considered indicators of low performance

The normalized confusion matrix for instrument detection for the best performing model, namely the MBOI+-model, is created in order to study the behavior of mis-classifications between instruments. For this matter, a successful detection is considered if the IoU metric of a predicted and a ground-truth bounding box is greater or equal to 0.5. The normalized confusion matrix is presented in Fig. 7.

The entries on the main diagonal of the presented confusion matrix indicate the percentage of correct classifications for a particular instrument class. It can be noted that the MBOI+-model is capable of correctly classifying more than 70% of the instances in the test set for 13 out of the 18 instruments present in our instrument set, indicating a promising performance. The mis-classifications could be attributed to insufficient examples in the training data or the reduced amount of SI images employed during the generation of the synthetic images created with the MBOI method. The classes with poor performance (entry on the main diagonal lower than 0.70) are Class 04, Class 08, Class 09, Class 10, Class 11. The problematic areas highlighted in red in the confusion matrix can be used to explain the poor performances in the aforementioned classes. In the case of the stick-like instruments, the off-diagonal entries indicate the presence of mis-classifications, also known as confusion, between Class 04 and Class 07. Similarly, in the case of the forceps (Class 08, Class 09, and Class 10), strong confusion can be noticed between the three instrument classes. In the case of the retractors (Class 11 and Class 12), a similar behavior can be observed. This supports our hypothesis i.e. it is hard for the detection algorithm to distinguish similar-looking instruments from one another.

The performance of the detection algorithm can potentially be improved by increasing the number of background and SI images employed during the generation of the MI synthetic images or simply by enlarging the amount of created data. This could allow the models to further learn the different features of similar-looking instruments and reduce the number of mis-classifications.

## Conclusions

In this work, we present a deep-learning-based approach for instrument detection under similar conditions to those of an actual operating room, i.e. multi-instrument images taken with a real scrub nurse under varying conditions.

We propose a novel data-augmentation technique, called MBOI, capable of automatically creating synthetic annotated images. By training on these artificial data, the Mask R-CNN algorithm is capable of achieving results comparable to the case in which real data is employed. This implies that our approach dramatically reduces the amount of annotating work required. The method can be applied to extend datasets that rely on annotations in the form of segmentation masks.

We create and make available for the scientific community the raw data required for the application of the MBOI method, for the specific case of the instruments of the surgical set for wisdom teeth extraction. We also include 369 real images of the same instrument sets and their corresponding segmentation masks.

A detection algorithm is trained and evaluated on the basis of these data for its future use in robotic scrub robot system. The algorithm achieved promising results, correctly classifying more than 70% of the instances in the test set for 13 out of the 18 instruments present in our instrument set.

Future experiments will focus on studying the relationship between the amount of raw data employed by the MBOI method, as well as the number of generated synthetic images, and the associated improvement of a model after training with these data. Additionally, 3D model-based data-augmentation approaches, such as the ones presented in [10, 18, 20], will be explored to further improve the performance of the instrument detection algorithm.
